# Personality prediction from task-oriented and open-domain human–machine dialogues

**DOI:** 10.1038/s41598-024-53989-y

**Published:** 2024-02-16

**Authors:** Ao Guo, Ryu Hirai, Atsumoto Ohashi, Yuya Chiba, Yuiko Tsunomori, Ryuichiro Higashinaka

**Affiliations:** 1 Graduate School of Informatics, Nagoya University, Nagoya, Japan; 2 NTT Communication Science Laboratories, NTT Corporation, Chiyoda, Japan

**Keywords:** Computer science, Software, Human behaviour

## Abstract

If a dialogue system can predict the personality of a user from dialogue, it will enable the system to adapt to the user’s personality, leading to better task success and user satisfaction. In a recent study, personality prediction was performed using the Myers–Briggs Type Indicator (MBTI) personality traits with a task-oriented human–machine dialogue using an end-to-end (neural-based) system. However, it is still not clear whether such prediction is generally possible for other types of systems and user personality traits. To clarify this, we recruited 378 participants, asked them to fill out four personality questionnaires covering 25 personality traits, and had them perform three rounds of human–machine dialogue with a pipeline task-oriented dialogue system or an end-to-end task-oriented dialogue system. We also had another 186 participants do the same with an open-domain dialogue system. We then constructed BERT-based models to predict the personality traits of the participants from the dialogues. The results showed that prediction accuracy was generally better with open-domain dialogue than with task-oriented dialogue, although Extraversion (one of the Big Five personality traits) could be predicted equally well for both open-domain dialogue and pipeline task-oriented dialogue. We also examined the effect of utilizing different types of dialogue on personality prediction by conducting a cross-comparison of the models trained from the task-oriented and open-domain dialogues. As a result, we clarified that the open-domain dialogue cannot be used to predict personality traits from task-oriented dialogue, and vice versa. We further analyzed the effects of system utterances, task performance, and the round of dialogue with regard to the prediction accuracy.

## Introduction

Recent studies have shown that user’s personality is significantly related to the performance of a dialogue system^1^ and that a dialogue system embedded with a personality matching that of its interlocutor can lead to better user satisfaction and perceived trustworthiness^2^. This means that if a dialogue system can predict the personality of a user from dialogue and adapt to the user’s personality, it can lead to better task success as well as user satisfaction.

Psychologists have proposed numerous personality theories over the years. Two mainstream personality traits, the Big Five personality traits and the Myers–Briggs Type Indicator (MBTI)^3^, have been extensively studied and predicted from human–human dialogues^4–6^. Recently, there has been an attempt to predict MBTI from a task-oriented human–machine dialogue using a neural-based end-to-end system^7^. However, previous research has only performed personality prediction from a specific task-oriented dialogue, it remains unclear whether personality prediction can be generally applied to human–machine dialogues and which personality traits can be accurately predicted.

To clarify this, we performed an extensive investigation into the prediction of personality traits from both task-oriented and non-task-oriented (open-domain) human–machine dialogues. We first recruited 378 participants and asked them to fill out four questionnaires. We then had them perform three rounds of human–machine dialogue with either a pipeline task-oriented dialogue system or an end-to-end task-oriented dialogue system. We also had another 186 participants do the same with an open-domain (chit-chat) dialogue using an end-to-end dialogue system. Following Fernau et al.’s^7^ research, we used the collected data to construct BERT-based models to predict the participants’ personality traits from the utterances. After that, we examined the effect of utilizing different types of dialogue on personality prediction by conducting a cross-comparison of the models trained from the task-oriented and open-domain dialogues. We further analyzed the effects of system utterances, task performance, and the round of dialogue with regard to the prediction accuracy. The contributions of this paper are as follows.

- This is the first comprehensive study that predicted 25 personality traits from both task-oriented and open-domain human–machine dialogues, with task-oriented dialogue using both pipeline and end-to-end systems.
- On the basis of crowdsourcing experiments, we demonstrated that the majority of the Big Five personality traits, the IOS, and several ATQ personality traits can be predicted from the open-domain human–machine dialogue, although Extraversion can be predicted equally well for both open-domain dialogue and pipeline task-oriented dialogue.
- From a cross-comparison of the models, we clarified that the prediction model trained from open-domain dialogue *cannot* be used to predict personality traits from task-oriented dialogue, and vice versa.

The rest of the paper is organized as follows. Related works on the influences of personality and personality prediction are detailed in the next section. “Approach” and “Data collection” describe the approach to personality prediction and the collection of experimental data. The experiments to examine personality prediction from task-oriented and open-domain dialogues are shown in “Experiments”. “Analyses” presents our analyses of factors that might have affected the prediction accuracy. Our conclusion and future work are described in “Conclusion and future work”.

## Related work

### Personality theories

Personality have been intensively developed and studies over the years. Currently, there are two mainstream personality theories describing the key personality on an individual^8^: the Big Five personality traits^9^ and the Myers–Briggs Type Indicator (MBTI)^10^. The Big Five personality traits were developed based on Goldberg’s lexical hypothesis that utilizes numerous English terms to describe inter-individual differences^11^. The MBTI was derived from Jung’s theory^12^, which categorizes personality into 16 types based on four binary dimensions: Extraversion–Introversion, Judgment–Perception, Thinking–Feeling, and Sensing–Intuition. In addition to these two mainstream theories, other theories have been proposed that describe specific facets or aspects of an individual, such as social or affect aspects. The Inclusion of Other in the Self (IOS) scale^13^, proposed as a social aspect of personality, is designed to measure an individual’s interpersonal closeness on seven scales. Kikuchi’s Scale of Social Skills (KISS-18)^14^ was developed to measure six key social skills of an individual. The Adult Temperament Questionnaire (ATQ)^15^ was designed to assess an individual’s personality with respect to affect.

### Personality and human–computer interaction

It is widely recognized that personality plays an important role in human–computer interaction (HCI)^16^. Lee et al. developed a social robot to talk with individuals and found that, individuals with high extraversion speak faster with a louder and higher pitched voice^17^. They also proposed the similarity-attraction principle, which assumes that individuals are more attracted to others who exhibit similar personalities^18^. Following this assumption, personality has been embedded into robots to enhance the user experience in HCI^19^. Mairesse and Walker developed a highly parameterizable dialogue generator that can generate utterances with either introverted or extraverted personalities^20^. Recently, Fernau et al. incorporated extraversion into a robot designed for job recommendations and observed increased user satisfaction^2^.

### Personality prediction from textual content

Personality manifests in the individual characteristics of behavior, cognition, and emotional patterns and has been extensively predicted from textual information^21,22^. In early studies, Pennebaker et al.^23^ built the Essays dataset containing 2468 anonymous essays tagged with the authors’ Big Five personality traits and analyzed the correlation between the linguistic styles of the authors and their personality traits. Argamon et al.^24^ extracted a set of lexical stylistic features related to function words and systemic functional grammar from the Essays dataset to predict Extraversion and Neuroticism. Mairesse et al.^25^ extracted the Linguistic Inquiry and Word Count (LIWC) features and Machine Readable Dictionary (MRC) psycholinguistic features from the Essays dataset and utilized support vector machines (SVMs) to predict the Big Five personality traits, achieving the average accuracy of 58%.

In addition to essays, users’ digital footprints (e.g., social media profiles and blog posts) have been widely utilized for personality prediction. The myPersonality dataset, which was collected by the Facebook App, contains users’ Facebook posts as well as the Big Five personality traits. Yu et al.^26^ predicted the Big Five personality traits from myPersonality by using neural network architectures such as fully connected (FC) networks, convolutional neural networks (CNN), and recurrent neural networks (RNN). Tandera et al.^27^ further utilized long short-term memory (LSTM) to better predict personality from myPersonality and achieved an average accuracy of 71% when using balanced data.

### Personality prediction from human–human dialogue

Dialogue has been increasingly adopted for personality prediction in recent years. Jurafsky et al.^5^ predicted a user’s interactional style (e.g., awkward, friendly, or flirtatious) from spoken conversation by using prosodic features and lexical features. Gjurković et al.^28^ built the PANDORA dataset by collecting Reddit comments from 10k users, among whom 1.6 k are labeled with the Big Five personality traits, and utilized BERT for personality prediction from these human–human dialogues. Khan et al.^29^ predicted the MBTI personality traits from an MBTI9k corpus^30^ of Reddit posts by fine-tuning the BERT model. Jiang et al. developed the FriendsPersona dataset from the public Friends TV Show Dataset and adopted both BERT and RoBERTa^31^ (as a robustly optimized BERT) models to predict the Big Five personality traits^32^. The best average prediction performance for the Big Five personality traits was achieved by RoBERTa, with a classification accuracy of 63%.

### Personality prediction from human–machine dialogue

As for human–machine dialogues, Fernau et al.^2^ found that a dialogue system embedded with a personality matching that of its interlocutor can lead to better user satisfaction and perceived trustworthiness. They also predicted the MBTI personality traits from a task-oriented human–machine dialogue for job recommendation using a BERT-based model and reported that the accuracy for Extraversion reached 69.17%^7^. Our motivation is similar to these studies in that we are interested in personality prediction for human–machine dialogues. We aim to obtain a general conclusion from the prediction of a wide coverage of personality traits (i.e., Big Five, IOS, KISS-18 and ATQ personality traits) based on two types of dialogue (task-oriented and open-domain dialogue) using different architectures (pipelines and end-to-end).

## Approach

A dataset of human–machine dialogue with personality traits is a prerequisite for achieving personality prediction from human–machine dialogues. To carry out a comprehensive study of personality prediction from human–machine dialogue, we first collected the relevant data and then built the model for personality prediction.

We selected the Big Five personality traits, which consist of five broad factors: Openness, Conscientiousness, Extraversion, Agreeableness, and Neuroticism. We also incorporate personality traits related to sociability and affect/temperament since previous studies have highlighted the importance of these two aspects in human–machine dialogue^33,34^. Specifically, we obtained personality traits from four prevalent personality questionnaires: (1) the Big Five Inventory (BFI-44)^35^, which contains 44 questions to assess the Big Five personality traits; (2) the Inclusion of Others in the Self (IOS) Scale^13^, which indicates how close a respondent feels to another person or group; (3) the Kikuchi’s Scale of Social Skills (KISS-18)^14^, which contains 18 questions to identify six social skills; and (4) the Adult Temperament Questionnaire (ATQ)^15^, which contains 77 questions to assess 13 user temperaments with regard to four dimensions.

The MBTI personality traits have attracted increasing attention in recent years, especially on social media platforms^36^. However, when considering the overlaps between the MBTI and the Big Five^37,38^, coupled with the broader acceptance of the Big Five in psychology^39,40^, we focus on the Big Five instead of the MBTI in this study.

To obtain general results, we focused on two mainstream types of human–machine dialogue: task-oriented and open-domain. Task-oriented dialogue aims to help users achieve specific goals, e.g., obtaining tourist information or making a restaurant reservation^41^. We utilized both pipeline and end-to-end (neural-based) task-oriented dialogue systems, as they represent the two typical types of dialogue systems^42^. Their distinct mechanisms for generating utterances make them ideal for testing the generality of personality prediction. A pipeline dialogue system has a modular and transparent pipeline in which each module can be implemented independently^43^. An end-to-end dialogue system is implemented by a neural-based sequence-to-sequence model that can generate system responses from a dialogue history^44^. In contrast to task-oriented dialogue, the purpose of open-domain dialogue is to keep the user engaged and chat about topics that he or she is interested in^45^. Considering the overwhelmingly better performance of end-to-end systems with open-domain dialogue^46^, we only used an end-to-end system for open-domain dialogue.

We first trained a set of personality prediction models from the training data of task-oriented and open-domain dialogues, and we then evaluated the models based on how accurately they predicted personality traits using the test data (“Results of prediction from task-oriented and open-domain dialogues”). In addition, we performed a cross-comparison to verify the generality of the models created from different types of dialogue (“Results of cross-comparison of models created from different types of dialogue”), e.g., whether the prediction model trained from open-domain dialogues can be used to predict personality from task-oriented dialogues. All experiments were conducted in conformity with the applicable rules and guidelines.

### Personality labeling

Following the majority of studies that predict personality in a classification manner^4,40^, we treat the personality prediction as a binary classification problem. A user’s personality trait is labeled as either “Low” or “High”: it is “Low” if its value is lower than the 50th percentile of all users’ trait values in the training and validation data, and “High” if its value is higher than the 50th percentile. Nevertheless, we have to admit that the binary results of predicted personality are easier to interpret, there is a loss of nuance that reduces the degree of statistical processability.

**Figure 1:**
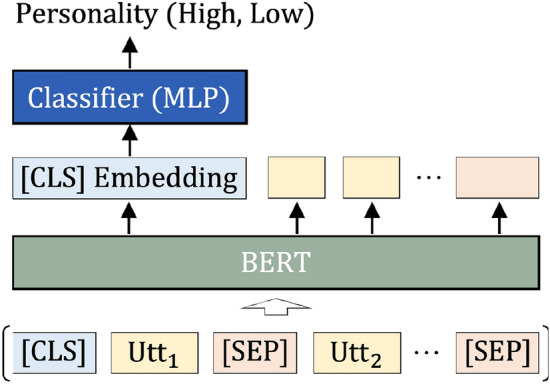
BERT-based model for personality prediction.

### BERT-based model

The BERT model has been widely used for personality prediction in recent studies and performs well^47^. In this study, we also train a BERT-based model to predict personality traits, as shown in Fig. 1. User utterances in a dialogue or both user and system utterances in a dialogue can be used as input for personality prediction. When utilizing user utterances, each user utterance within a dialogue is sequentially concatenated with a special [SEP] token. A special [CLS] token is added to the beginning of the sequence for personality classification to output a binary label for a personality trait. When using both user and system utterances, a special [USR] or [SYS] token is added in front of each user or system utterance instead of the [SEP] token.

### Evaluation metric and baseline

As we use 10-fold cross-validation in “Model configuration for personality prediction”, the test data in each fold may be imbalanced. To evaluate the prediction performance of the model from the data with imbalanced personality labels, we selected balanced accuracy as an evaluation metric. Balanced accuracy is calculated by averaging the true positive rate (TPR) and true negative rate (TNR)^48^:

1 $$\begin{aligned} \text {Balanced Accuracy }= \frac{\textrm{TPR} + \textrm{TNR}}{2}. \end{aligned}$$

As a baseline, we selected a majority-based classifier that uses the most frequent personality label occurring in the training and validation data as its prediction. The balanced accuracy of the baseline is constantly 50% since we have two labels.

## Data collection

The data collection experiment was designed as a Human-Intelligence Task (HIT) with the aim of obtaining participants’ personality traits and their human–machine dialogues on the Amazon Mechanical Turk (AMT) crowdsourcing platform. The HIT was composed of two parts: filling out personality questionnaires and engaging in three rounds of conversation with a pipeline task-oriented dialogue system, an end-to-end task-oriented dialogue system, or an open-domain dialogue system. We collected approximately 600 participants’ personality traits and their three rounds of dialogue with one of the three dialogue systems. The HIT was approved by the ethical review committee of our organization.

To guarantee the quality of the collected dialogues, we limited the participants’ regions to English-speaking countries and set the HIT qualifications to (1) HIT accomplishment number greater than 100 and (2) HIT approval rate greater than 95%. Every participant who finished the HIT received a payment of $10. The HIT accomplishment number is the total number of HITs completed by a worker on AMT^49^. This qualification restricts tasks to participants with high levels of experience based on their past work. Each participant was only permitted to complete the HIT for one of the three dialogue systems.

### Collection of task-oriented dialogues

MultiWOZ 2.1^50^, which is a large-scale human–human dialogue corpus spanning several tourist information consulting tasks, was selected to build the dialogue systems for collecting task-oriented dialogues. The task domains are restaurant, hotel, attraction, taxi, train, hospital, and police. Note that we did not use the hospital or police domains because they contain significantly fewer slots and values compared to the other five. Before starting, participants were required to read the description of the assigned tasks carefully. This description included the goal that the user needed to achieve, e.g., book an Indian restaurant with a table for three people. We implemented the pipeline and end-to-end dialogue systems as follows.

- Pipeline dialogue system: the best-performing dialogue system implemented by the ConvLab-2 toolkit^51^ was used as the task-oriented dialogue system. It has a pipeline architecture consisting of a BERT-based natural language understanding (NLU) module, a rule-based dialogue state tracking (DST) module, a rule-based policy, and a natural language generation (NLG) module using templates.
- E2E dialogue system: the Simple Task-Oriented Dialogue (SimpleTOD)^52^ with End-to-End (E2E) structure was selected. SimpleTOD is a GPT2-driven language model fine-tuned for MultiWOZ dialogues, where the model incrementally generates a belief state, system actions, and system response conditioned on the dialogue history (past user and system utterances). Since the best hyper-parameter for SimpleTOD is undisclosed, we trained the model using the public source code published on GitHub with different hyper-parameter configurations and selected the most optimized model (see “Model configuration for personality prediction”).

Four levels of task difficulty (*easy*, *normal*, *hard*, and *very hard*) were defined for the task-oriented dialogues. The task, which ranges in difficulty from *easy* to *very hard*, involves one to four domains with a total of five to 20 slots. Here, a slot in a task-oriented dialogue system represents a key-value pair of information to be inquired from the dialogue system^53,54^. In general, hardness is related to the number of slots; an increased number of slots requires a higher communicative effort from the participant, which subsequently increases the difficulty of the task.

An example of a task description with easy difficulty in the train domain is presented as follows: *“You are looking for a train. The train should depart from Kings Lynn and go to Cambridge. The train should leave on Wednesday after 21:00. Once you find a train, make sure you get the train ID and travel time.”*

An example of a task description with hard difficulty is presented as follows. *“You are looking for a place to stay. The hotel should have a star of 3. The hotel should be in the moderate price range. The hotel should include free parking. Once you find a hotel, make sure you get the hotel type. You are also looking for a train. The train should depart from Cambridge and go to Stevenage. The train should leave after 10:45 on Tuesday. Once you find a train, make sure you get the travel time, and price.”*

For the pipeline dialogue system, we randomly generated a dialogue task with a *hard* difficulty containing three domains with 15 slots or a *very hard* difficulty containing four domains with 20 slots. Participants were required to finish a dialogue task within 30 utterances. Considering that the task performance of the E2E dialogue system is typically worse than that of the pipeline system^51^, a dialogue task with a *normal* difficulty containing two domains with ten slots or a *hard* difficulty with 15 slots were randomly assigned for the E2E task-oriented dialogue system. Participants were required to finish a dialogue task within 20 utterances. The domains for both the pipeline and E2E task-oriented dialogues were randomly generated by the Goal Generator function of ConvLab-2. We determined the difficulty of the task and the maximum utterances for the pipeline and the E2E dialogue systems through a small-scale internal experiment. The task success rates for the pipeline dialogue system, from easy to very hard, were 93.3%, 80%, 82%, and 60%, respectively. For the E2E dialogue system, the task success rates from easy to hard were 60%, 55%, and 40%. On the basis of these results, we selected the *very hard* difficulty for the pipeline system and the *normal* difficulty for the E2E system. In total, we collected data from 204 participants for the pipeline task-oriented dialogue system, and from 205 participants for the E2E task-oriented dialogue system.

### Collecting open-domain dialogues

A Transformer sequence-to-sequence conversational model named BlenderBot^55^ was used as the open-domain dialogue system for its good conversational ability. We used the distilled BlenderBot model with 400M parameters to take advantage of its reasonable response time (around one second per utterance). The implementation was adopted from ParlAI^56^. Participants were asked to have a leisurely chat with the system for 20 utterances. We assigned a randomly generated persona (about five representative statements such as “I am a vegetarian. I like swimming.”^57^) to the system as its background for each dialogue. In total, we collected data from 201 participants for the open-domain dialogue system.

### Data statistics

To reduce the effect of noise, we first filtered the collected data on the basis of two criteria: (1) if the participant finished three rounds of dialogue in less than 5 min and (2) if any round of dialogue had a user vocabulary less than 10. Here, vocabulary refers to the unique words in all user utterances within a dialogue, and is formulated as

2 $$\begin{aligned} \textrm{Vocabulary} = \textrm{Token}({[\text {utt}_{1}||\ldots ||\text {utt}_{\textrm{N}}]}), \end{aligned}$$

where $\textrm{Token}$ is a function that counts the unique words (as tokens) from its input dialogue with $\textrm{N}$ utterances $[\text {utt}_{1}||\ldots ||\text {utt}_{\textrm{N}}]$, and || denotes the concatenation operation. Any seemingly low-quality dialogues from non-engaged participants were filtered out by the two criteria.

The statistics of the collected data after filtering are listed in Table 1. In addition to vocabulary, we calculated the lexical diversity by the ratio of vocabulary to the total number of words:

3 $$\begin{aligned} \text {Lexical Diversity} = \frac{\textrm{Vocabulary}}{{\textrm{Word}}([\text {utt}_{1}||\ldots ||\text {utt}_{\textrm{N}}])}, \end{aligned}$$

where $\textrm{Word}$ is a function that counts the total number of words from its input utterances $[\text {utt}_{1}||\ldots ||\text {utt}_{\textrm{N}}]$.

We can see in Table 1 that the pipeline task-oriented dialogues had a moderate success rate (47%), whereas the E2E task-oriented dialogues had a lower success rate (28%), as suggested by the research conducted by Zhu et al.^51^ It is also clear that the open-domain dialogues had the highest number of vocabulary and lexical diversity for both user and system utterances, probably because there was no domain restriction in the open-domain dialogues. Examples of the three types of dialogue are shown in Table 2.

**Table 1:** Statistics of collected data after filtering out low-quality data.

Data statistics	Task-pipeline	Task-E2E	Open-domain
Number of participants	179	199	186
Number of dialogues	537	597	558
Average user words/utterance	7.8	6.9	9.2
Average system words/utterance	18.1	16.0	19.7
User lexical diversity	0.30	0.35	0.42
System lexical diversity	0.21	0.19	0.27
Average user response time	26.0 s	24.3 s	24.6 s
Success rate/dialogue	47%	28%	N/A
Average user vocabulary/dialogue	70.8	58.1	90.2
Average system vocabulary/dialogue	121.4	82.2	155.1

Task-pipeline and Task-E2E denote pipeline task-oriented dialogue and E2E task-oriented dialogue.

**Table 2:** Excerpts of pipeline task-oriented dialogue, E2E task-oriented dialogue, and open-domain dialogue taken from crowdsourced data.

Task-pipeline	
Usr: Hello, I am looking for local tourist attractions in the east of Cambridge.	
Sys: We’ve 10 options, anything in particular you are looking for? Cherry hinton hall and grounds looks good, would you like to head there?	
Usr: Can you tell me a bit about it?	
Sys: They are located at pool way, whitehill road, off newmarket road.	
Usr: I am also looking for a moderately priced restaurant that serves gastropub food in the centre of Cambridge	
Sys: How does the the cow pizza kitchen and bar sound? That is a gastropub restaurant. It is in the centre area	
Usr: Sounds good! What is the phone number and address for cow pizza kitchen and bar?	
Sys: The restaurant’s phone number is 01223308871. Their address is Corn Exchange Street. It is in the centre area	
Task-E2E	
Usr: Can you help find a local restaraunt Nandos City Centre?	
Sys: I am sorry, could you rephase your sentence?	
Usr: I am trying to find the postal code for Nandos City Centre?	
Sys: Yes, the postal code is cb12lj. Is there anything else i can help you with?	
Usr: does lensfield hotel offer free parking?	
Sys: Yes, it does offer free parking	
Usr: Thank you. Have you booked me at lensfield hotel for 7 people?	
Sys: Sure, I have it booked for you. Is there anything else I can help you with?	
Open-domain	
Usr: Hi, how’s your day going?	
Sys: It is going well. I just got back from a walk with my dog. How is your day?	
Usr: My day has gone well, somewhat boring	
Sys: I’m sorry to hear that. Do you have any hobbies to keep you occupied?	
Usr: I like to play on the computer and you?	
Sys: I like playing video games as well. What kind of computer do you have? I have a gaming computer	
Usr: I like to play League of Legends	
Sys: I’ve never played that, but I’ve heard good things. What do you like to do for fun?	

## Experiments

Using the collected data, we first conducted an experiment to assess the personality prediction from task-oriented and open-domain dialogues, where the task-oriented dialogue system was developed using pipeline and end-to-end architectures. Then, we performed a cross-comparison of the models created from task-oriented and open-domain dialogues to verify the generality of such models.

### Model configuration for personality prediction

The BERT-based model for personality prediction was implemented using Simple Transformers, which is a library built on Hugging Face’s Transformers library^58^. We chose the common “BERT-base-uncased”^47^ model for the classification of dialogues without system utterances involved as input (see “Impact of system utterances”; we found that system utterances do not benefit the personality prediction). We limited the input dialogue to a maximum token number of 512 and trained the model with a batch size of 8, optimized by AdamW set to the learning rate of 2e−5 with early stopping and the patience of 3. We set the maximum number of epochs to 20 and validated the model every 25 training steps.

**Figure 2:**
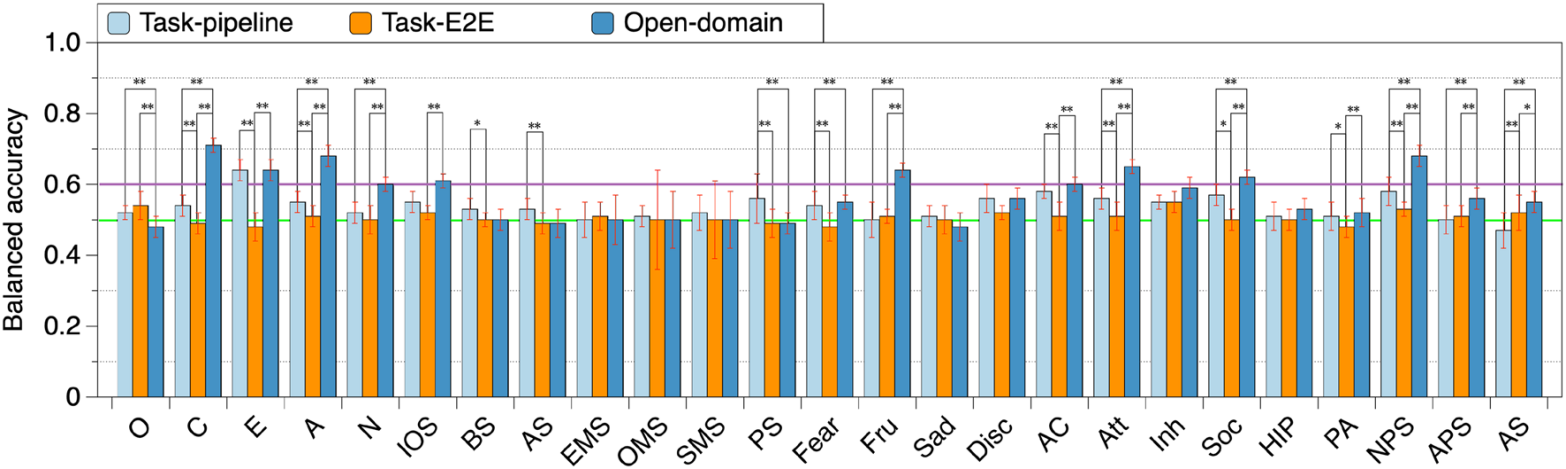
Personality prediction from task-oriented and open-domain dialogues with user utterances only. *O* openness, *C* conscientiousness, *E* extraversion, *A* agreeableness, *N* neuroticism, *Fru* frustration, *Disc* discomfort, *AC* activation control, *Att* attentional control, *Inh* inhibitory control, *Soc* sociability, *HIP* high intensity pleasure, *PA* positive affect, *NPS* neutral perceptual sensitivity, *APS* affective perceptual sensitivity, *AS* associative sensitivity. Error bars indicate standard deviation (*$p<0.05$, **$p<0.01$).

### Results of prediction from task-oriented and open-domain dialogues

Figure 2 shows the results of personality prediction using the “BERT-base-uncased” model separately fine-tuned by pipeline task-oriented (Task-pipeline), E2E task-oriented (Task-E2E), and open-domain (Open-domain) dialogues. Note that only user utterances were utilized for the personality prediction. We used 10-fold cross-validation. Specifically, the participants’ dialogues were first shuffled by a random seed and partitioned into ten equally sized folds. A single fold was retained for validation while another single fold was assigned for test. The remaining eight folds were used for training. There was no overlapping among participants in the training/validation/test dialogues. For each fold used as test data, the prediction result was calculated using the corresponding trained model. To ensure the reliability and stability of the results, we repeated the cross-validation process ten times, each time using a different random seed to shuffle the dialogues. The balanced accuracy utilized for prediction performance comparison (depicted in each bar of Fig. 2) was calculated by averaging the balanced accuracies of ten cross-validation trials.

A non-parametric t-test with two-stage false discovery rate correction ($\alpha = 0.05$) was carried out to assess the statistical significance of the prediction results for a certain personality trait among the three types of dialogue. Here, we compared the accuracies averaged over the ten cross-validation trials. As Fig. 2 shows, the prediction accuracies with the open-domain dialogue for Conscientiousness (71%), Agreeableness (68%), Neuroticism (60%), IOS (61%), Frustration (64%), Activation Control (60%), Attentional Control (65%), Sociability (62%), and Neural Perceptual Sensitivity (68%) were better than those with the task-oriented dialogue. In addition, Extraversion could be predicted equally well for both open-domain dialogue (64%) and pipeline task-oriented dialogue (64%).

We compared the accuracy of our personality prediction to that of previous studies. When we look at the result of prediction from another human–machine dialogue with a different dialogue task (job recommendation), our prediction of Extraversion (with 64% of dialogue correctly classified) reached the same level of accuracy as those from the research by Fernau et al.^7^ (with 69% of dialogue correctly classified by a BERT-based model), indicating that Extraversion can be generally predicted from the different task-oriented human–machine dialogues. Examining the results of Big Five personality predictions from speech transcripts^59^ (textual data), we find that the accuracy of Conscientiousness, Extraversion, Agreeableness, and Neuroticism predicted from the human–machine dialogue in our study (70%, 64%, 68%, and 59%) reached the same level of accuracy as those predicted from speech (61%, 61%, 66%, and 61%), although the accuracy of Openness (55%) predicted from the human–machine dialogue was lower than that from speech (66%). This may be due to the fact that individuals with high Openness tend to exhibit emotional expressiveness, which can easily be reflected in speech but may not be as evident in textual dialogue.

**Table 3:** Cross-comparison of models created from task-oriented and open-domain dialogues.

Test	Conscientious			Extraversion			Agreeableness			Neuroticism			IOS		
Train	P	E	O	P	E	O	P	E	O	P	E	O	P	E	O
P	0.55	0.53	0.57	0.66	**0.61**	0.62	0.55	0.52	0.54	0.52	0.49	0.49	0.56	0.50	0.57
E	0.53	0.49	0.48	0.55	0.48	0.55	0.49	0.51	0.45	0.50	0.51	0.48	0.51	0.51	0.55
O	0.52	0.50	0.71	0.52	0.50	0.65	0.51	0.47	0.70	0.50	0.48	0.61	0.56	0.50	0.60
P+E	0.54	0.51	0.53	0.52	0.49	0.61	0.54	0.51	0.52	0.51	0.50	0.49	0.54	0.50	0.57
P+O	*0.56*	0.53	0.70	0.65	0.51	0.65	0.55	0.50	0.68	0.50	0.48	0.58	0.56	0.50	0.58
E+O	0.54	0.51	0.68	0.61	0.50	0.63	0.53	0.51	0.66	0.49	0.50	0.57	0.55	0.52	0.59
P+E+O	*0.56*	0.52	0.67	0.64	0.50	0.63	0.53	0.50	0.65	0.50	0.49	0.57	0.55	0.51	0.59

Test	Frustration			Act. Control			Att. Control			Sociability			NPS		
Train	P	E	O	P	E	O	P	E	O	P	E	O	P	E	O
P	0.50	0.51	0.53	0.57	0.52	0.53	0.55	0.51	0.61	0.57	0.49	0.57	0.62	0.52	0.62
E	0.53	0.50	0.52	0.52	0.49	0.51	0.52	0.50	0.51	0.51	0.49	0.53	0.49	0.52	0.48
O	0.52	0.53	0.60	0.52	0.52	0.63	0.54	0.51	0.68	0.57	0.50	0.62	0.58	0.50	0.69
P+E	0.51	0.51	0.52	0.57	0.52	0.55	0.55	0.52	0.58	0.53	0.50	0.57	0.58	0.52	0.57
P+O	0.50	0.52	0.59	0.56	0.53	0.62	*0.57*	0.50	0.68	*0.58*	0.49	0.61	0.61	0.50	0.69
E+O	0.53	0.51	0.60	0.53	0.53	0.60	0.55	0.52	0.67	0.55	0.49	0.60	0.56	0.51	0.68
P+E+O	0.51	0.53	0.59	0.56	0.53	0.61	0.54	0.53	0.67	0.57	0.50	0.59	0.61	0.50	0.68

*P* pipeline task-oriented dialogue, *E* E2E-based task-oriented dialogue, *O* open-domain dialogue, *Act. Control* action control, *Att. Control* attention control, *NPS* neutral perceptual sensitivity.

### Results of cross-comparison of models created from different types of dialogue

To clarify whether different types of dialogue can benefit the prediction of user personality from a certain type of dialogue, we performed cross-comparison of the models created from various types of dialogue. For this purpose, we first shuffled the three types of dialogue datasets (i.e., pipeline task-oriented dialogue, E2E task-oriented dialogue, and open-domain dialogue) and split them into training, validation, and test sets with a split ratio of 8:1:1. Similar to “Results of prediction from task-oriented and open-domain dialogues”, only user utterances were utilized for the personality prediction. We then fine-tuned the “BERT-base-uncased” models using the same type of training and validation sets and evaluated them with different test sets. Note that, for the purpose of comparing the models, we fixed the training, validation, and test sets; we did not perform cross-validation on models as described in “Results of prediction from task-oriented and open-domain dialogues” . To maintain the reliability and stability of the comparison, each accuracy result was obtained by averaging the results of 100 trials with different random seeds for shuffling the datasets to create training, validation, and test sets for each trial.

Table 3 presents the prediction accuracy of ten personality traits that can be reasonably predicted in Fig. 2 (with an accuracy equal to or greater than 60% predicted from any type of dialogue). We separately trained prediction models using a certain training set of “P” (pipeline task-oriented dialogue), “E” (E2E-based task-oriented dialogue), and “O” (open-domain dialogue). We then tested their prediction accuracy using the respective test sets of “P”, “E”, and “O”. Results in bold indicates results using training data and test data from different types of dialogue, where the result is better than that from the same type of dialogue. Note that the results below 0.55 are not in bold, as we consider such results to indicate low prediction performance. We found that the model trained with different types of dialogue did not benefit the prediction, except for the prediction of Extraversion, where “P” served as training data and “E” as the test data.

In addition to training a model using a single type of dialogue (i.e., “P”, “E”, or “O”), we also trained the model using additional types of dialogue: “P+E” (pipeline task-oriented dialogue and E2E-based task-oriented dialogue), “P+O” (pipeline task-oriented dialogue and open-domain dialogue), “E+O” (E2E-based task-oriented dialogue and open-domain dialogue), and “P+E+O” (pipeline task-oriented dialogue, E2E-based task-oriented dialogue, and open-domain dialogue). Italic values indicate better results when using additional types of dialogue. As we can see, the use of open-domain dialogue as additional training data provided negligible improvement in the prediction for the pipeline task-oriented dialogue. We consider that the expression of personality in task-oriented dialogue differs from that in open-domain dialogue due to the content differences. As a result, we conclude that, although the content of the open-domain dialogue may be diverse and cover a wide range of topics, such dialogue cannot be used to improve the prediction accuracy of a model built from task-oriented dialogue.

## Analyses

In our further analyses, we performed predictions using system utterances and predictions from different rounds of dialogue. We also examined the predictions derived from task-success and task-failure dialogues. We followed the same procedure as described in “Results of prediction from task-oriented and open-domain dialogues” to obtain prediction results and assess statistical significance. Our findings revealed only a limited impact of system utterances, dialogue rounds, and task-success/task-failure dialogues, leading to some important conclusions.

**Figure 3:**
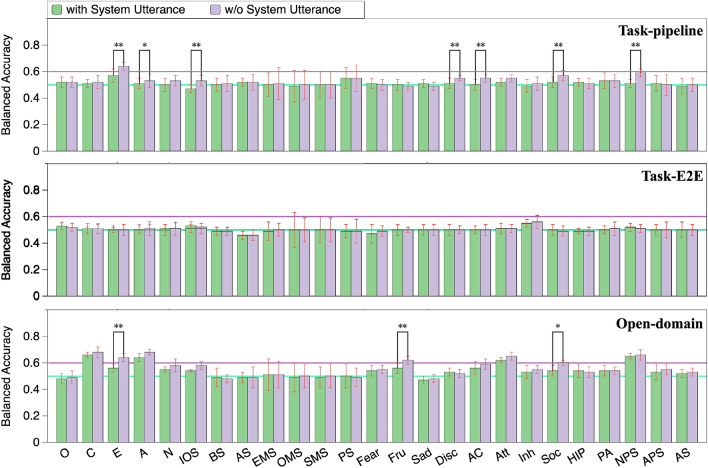
Personality prediction from task-oriented and open-domain dialogues with and without system utterances involved (*$p<0.05$, **$p<0.01$).

### Impact of system utterances

When we utilize both user and system utterances to predict personality, we can include user reactions to system utterances, which can possibly lead to better prediction accuracy. To verify this, we compared the personality prediction from only user utterances and that from both user and system utterances, as shown in Fig. 3. Since the majority of model inputs, which consist of both user and system utterances, exceeded the maximum number of tokens supported by “BERT-base-uncased” (512 tokens), we utilized the “Longformer-base-4096”, which can handle inputs of up to 4096 tokens, to fine-tune the model for inputs both with and without system utterances. Note that, in our study, both the “BERT-base-uncased” and “Longformer-base-4096” were able to cover all of their corresponding inputs.

We can see here that the accuracy for each personality trait predicted from both user and system utterances was generally worse than that from only user utterances: in other words, system utterances negatively affected the prediction accuracy. This is probably because the personality traits are exhibited sufficiently in user utterances, while system utterances act as noise that misleads the prediction of users’ personality traits.

**Figure 4:**
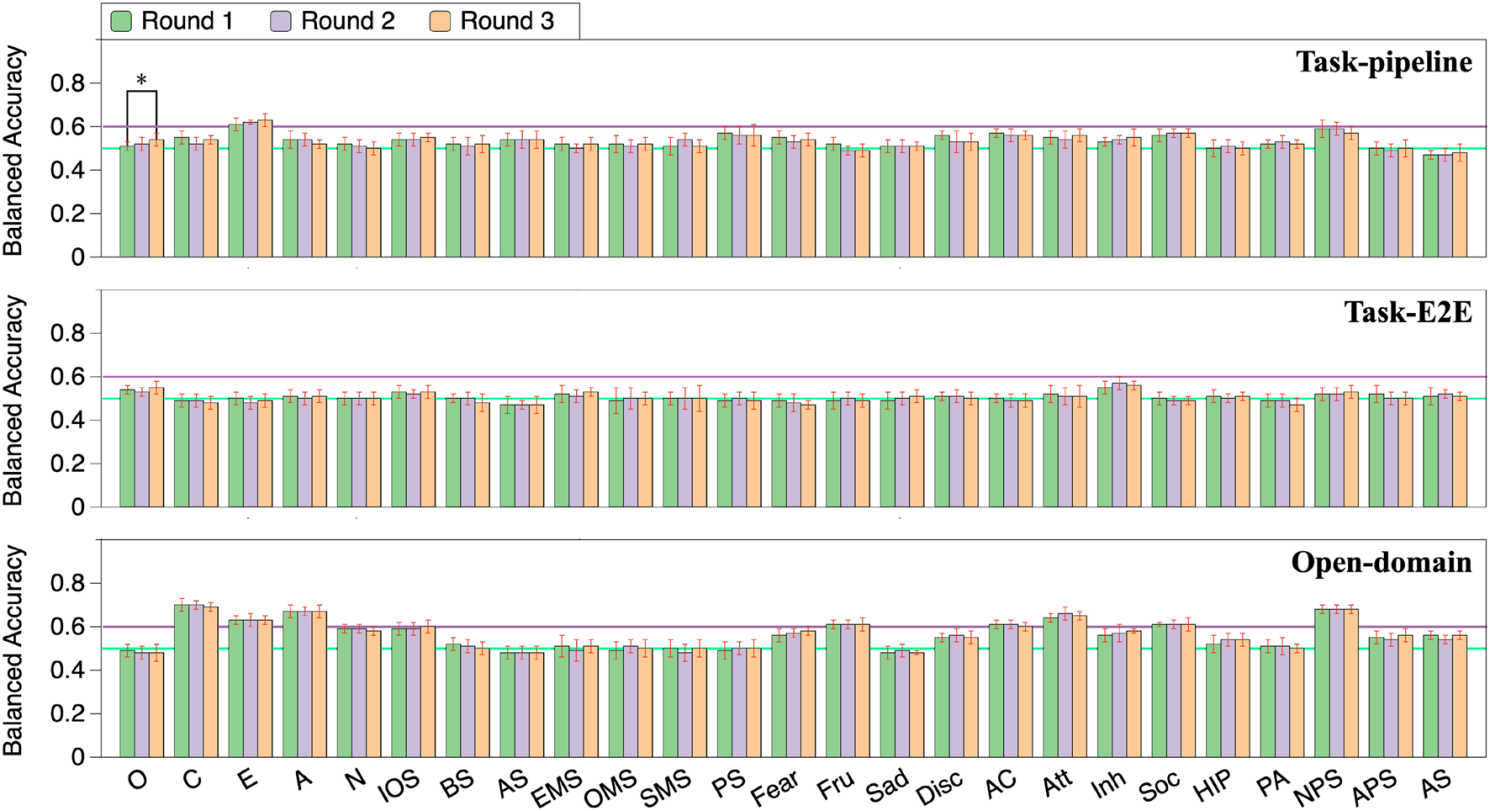
Personality prediction from different rounds of dialogue (*$p<0.05$).

**Figure 5:**
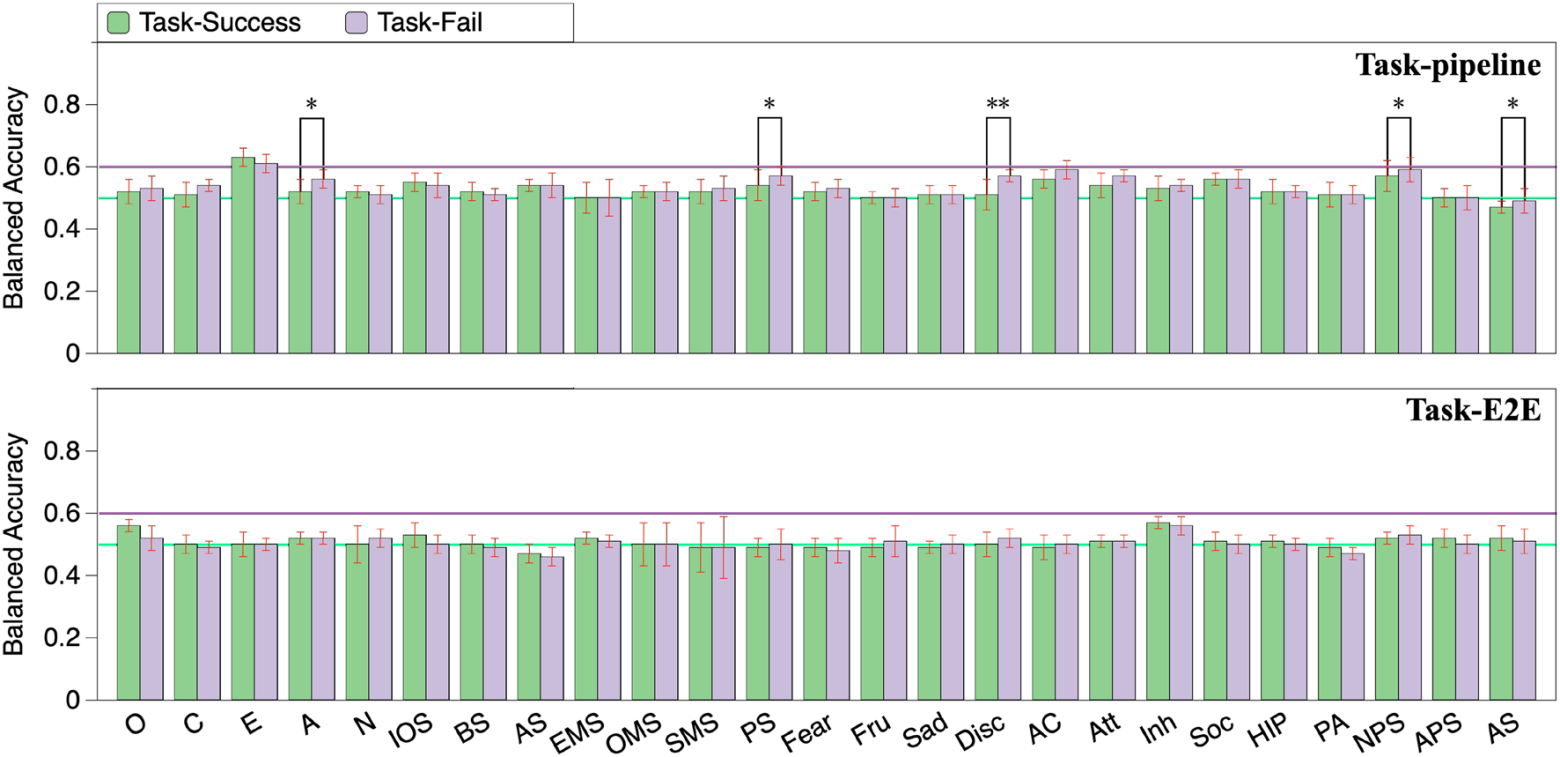
Personality prediction from task-success and task-failure dialogues (*$p<0.05$).

### Analysis of prediction from different rounds of dialogue

The dialogue round (indicating how many times a user has had conversations with a system) may have an effect on personality prediction, as the user may change his/her dialogue features (e.g., vocabulary and utterance length) as the dialogue progresses, exposing different personality traits in the process. To verify this, we analyzed the personality prediction from all rounds, the first round, the second round, and the third round of dialogue (without system utterances involved) by fine-tuning the “BERT-base-uncased” model. The prediction results are presented in Fig. 4, where the upper graph shows the results from the pipeline task-oriented dialogue system, the middle graph from the E2E task-oriented dialogues, and the lower graph from the open-domain dialogues. We can see here that there were few cases with statistical significance. These results suggest that the prediction accuracy remains unchanged across different turns of dialogue. This may be a positive finding because only a first-time dialogue is sufficient for personality prediction.

### Analysis of predictions from dialogues with different task performances

Users typically exhibit different expressions in dialogues with different task performances (i.e., task-success and task-failure dialogues). For example, they may put more effort into expressing their desires or feelings in task-failure dialogues compared to task-success dialogues. As a result, personality traits might be more predictable in dialogues with specific task performances. To verify this, we separately analyzed the personality prediction for task-success and task-failure dialogues (without system utterances involved). The prediction results are presented in Fig. 5, where the upper graph shows the results from the pipeline task-oriented dialogues and the lower graph from the E2E task-oriented dialogues. We can observe that the prediction accuracy for personality traits shows almost no difference between task-success and task-failure dialogues. These results suggest that task performance does not need to be considered when predicting personality traits from task-oriented dialogues.

**Figure 6:**
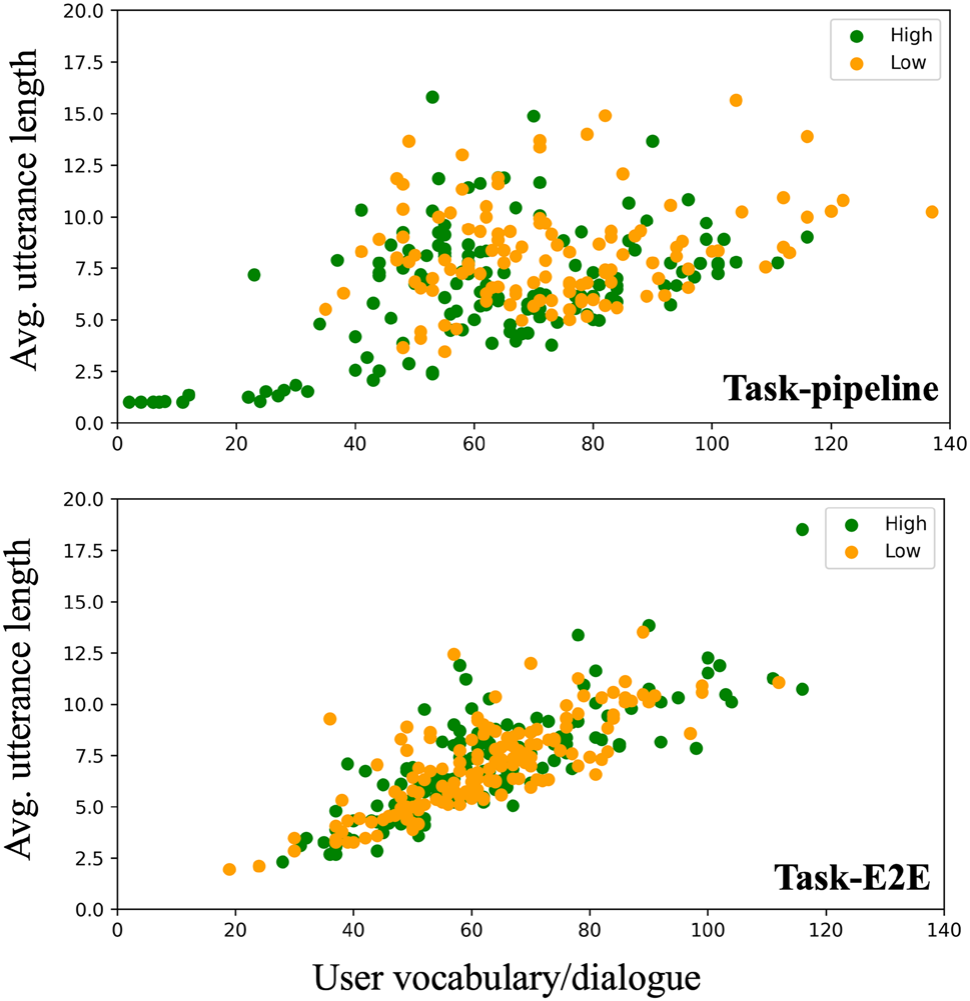
Scatter plots of user vocabulary and average utterance length from dialogues with high and low Extraversion.

### Analysis of the cause of low prediction accuracy for E2E task-oriented dialogues

To clarify the cause of the low prediction accuracy for Extraversion from the E2E task-oriented dialogues, we extracted user vocabulary (as defined in Eq. 2) and average utterance length from the pipeline and E2E task-oriented dialogues, as our prior work has indicated that both are related to Extraversion^1^.

Scatter plots of user vocabulary and average utterance length from dialogues with high and low Extraversion are shown in Fig. 6. As we can see at the top of the figure, users with high Extraversion used short utterances with low vocabulary when chatting with the pipeline dialogue system. As for the E2E dialogue system (bottom of the figure), the user vocabulary and average utterance length of the users with high or low Extraversion were almost the same. We can also see that both the user vocabulary and average utterance length for the E2E task-oriented dialogues exhibited smaller variance compared to those for the pipeline task-oriented dialogues.

Considering the low ability of the E2E dialogue system implemented with SimpleTOD (28% success rate), we conclude that such an E2E system is incapable of understanding user utterances very well, which in turn limits the user’s free expression toward the dialogue system, thus leading to a low prediction accuracy. This analysis leads us to believe that the task performance of the dialogue system is also an important feature in achieving high-quality personality prediction.

## Conclusion and future work

Our objective in this work was to derive a general conclusion on the prediction of personality traits from human–machine dialogue across different system types and architectures. To this end, we collected personality-labeled dialogues using a pipeline task-oriented dialogue system and an E2E task-oriented dialogue system. We also collected dialogues using an open-domain dialogue system. We then built a BERT-based model to predict 25 personality traits of users from the user utterances of a dialogue.

The results showed that the prediction accuracy was better with the open-domain dialogue than with the task-oriented dialogue, although Extraversion could be predicted from a pipeline task-oriented dialogue at the same level. We also performed a cross-comparison of models created from task-oriented and open-domain dialogues and found that the prediction models had low generality and could not be used to accurately predict personality from different types of dialogue. Our findings lead us to recommend that initiating an open-domain dialogue at the beginning of a conversation is the most effective way to develop a dialogue system that can predict and adapt to user personality. When one is focused on Extraversion, it may also be possible to predict user personality from task-oriented dialogue with a pipeline architecture. We further investigated the effects of using system utterances and prediction from different rounds of dialogue and found that the system utterances and dialogue round had only a limited effect on the personality prediction. We also had an indication that the low ability of the implemented E2E dialogue system (SimpleTOD) limited the prediction of personality.

Regarding the 60% performance of personality prediction in this study, we acknowledge that it is not a high value. However, we believe this is presumably the best we can attain using current techniques. This is backed by previous work as well as our empirical work in the current study. Our analysis of the cause of low prediction accuracy for E2E task-oriented dialogues indicates that the accuracy is related to the limited vocabulary of the dialogue system. When a dialogue system has a limited vocabulary, it is incapable of understanding user utterances very well, which in turn limits the user’s free expression toward the system. However, the 60–70% accuracy of personality prediction in prior studies related to human–human dialogues suggests that there might be other factors influencing the personality prediction. For example, an individual’s personality might not be fully expressed due to factors such as interpersonal relationships or the communication style of their interlocutors.

Achieving 60% accuracy in personality prediction would enable a dialogue system to adapt, to a certain extent, to the personality of its interlocutor, thereby improving user satisfaction. However, there is a risk of decreased user satisfaction when users’ personalities are misclassified. To improve the accuracy of predicted user personality, it will be necessary to incorporate other methods.

Much additional study remains as future work. First, other classification models (e.g., RoBERTa^31^ or DeBERTa^60^) should be considered to improve prediction accuracy. Second, we want to perform more rigorous analyses of the results we obtained in the experiments, such as incorporating additional word-related features (e.g., part-of-speech tag^61^ and Linguistic Inquiry and Word Count^62^) to examine the cause of low prediction accuracy for E2E task-oriented dialogues. Third, we also want to conduct a more comprehensive analysis of the influence of system responses to thoroughly examine their possible effects. Fourth, other dialogue tasks (e.g., job recommendation^7^ and car assistant^63^) and a wider variety of dialogue systems (e.g., dialogue system based on large language models^64^) should be adopted for a more comprehensive evaluation. Fifth, regarding the factors influencing personality prediction, we would like to investigate the causes of the accuracy limitation in personality prediction. Last but not least, since dialogue systems can achieve higher satisfaction when their personality traits align with those of their interlocutors, we aim to assess the personality traits of interlocutors from human–machine dialogues and subsequently to incorporate the corresponding personality traits into the dialogue system.

## Data Availability

The dataset and code used in the current study are available from the corresponding author upon reasonable request.

## References

[1] Guo, A. *et al.* Influence of user personality on dialogue task performance: A case study using a rule-based dialogue system. In *Proceedings of the 3rd Workshop on Natural Language Processing for Conversational AI*, 263–270 (2021).

[2] Fernau, D., Hillmann, S., Feldhus, N., Polzehl, T. & Möller, S. Towards personality-aware chatbots. In *Proceedings of the 23rd Annual Meeting of the Special Interest Group on Discourse and Dialogue*, 135–145 (2022).

[3] Furnham A (2020). Myers–Briggs type indicator (MBTI). Encyclop. Personal. Individ. Differ..

[4] Vinciarelli A, Mohammadi G (2014). A survey of personality computing. IEEE Trans. Affect. Comput..

[5] Jurafsky, D., Ranganath, R. & McFarland, D. Extracting social meaning: Identifying interactional style in spoken conversation. In *Proceedings of the 2009 Annual Conference of the North American Chapter of the Association for Computational Linguistics*, 638–646 (2009).

[6] Chen, Y.-H. & Choi, J. D. Character identification on multiparty conversation: Identifying mentions of characters in TV shows. In *Proceedings of the 17th Annual Meeting of the Special Interest Group on Discourse and Dialogue*, 90–100 (2016).

[7] Fernau, D., Hillmann, S., Feldhus, N. & Polzehl, T. Towards automated dialog personalization using MBTI personality indicators. In *Proceedings of Interspeech*, 1968–1972 (2022).

[8] McCrae, R. R. & Costa, P. Sage handbook of personality theory and assessment: Volume 1 personality theories and models. *Boyle, G. J., et al. (eds)* 273–294 (2008).

[9] Roccas S, Sagiv L, Schwartz SH, Knafo A (2002). The Big Five personality factors and personal values. Pers. Soc. Psychol. Bull..

[10] Myers IB (1962). The Myers-Briggs Type Indicator: Manual (1962).

[11] Goldberg LR (2013). An alternative “description of personality”: The Big-Five factor structure. Personality and Personality Disorders.

[12] Jung C, Beebe J (2016). Psychological Types.

[13] Aron A, Aron EN, Smollan D (1992). Inclusion of other in the self scale and the structure of interpersonal closeness. J. Pers. Soc. Psychol..

[14] Takahashi J, Tamaki K, Yamawaki N (2013). Autism spectrum, attachment styles, and social skills in university student. Creat. Educ..

[15] Evans DE, Rothbart MK (2007). Developing a model for adult temperament. J. Res. Pers..

[16] Pocius KE (1991). Personality factors in human-computer interaction: A review of the literature. Comput. Hum. Behav..

[17] Lee KM, Peng W, Jin S-A, Yan C (2006). Can robots manifest personality?: An empirical test of personality recognition, social responses, and social presence in human-robot interaction. J. Commun..

[18] Nass C, Lee KM (2001). Does computer-synthesized speech manifest personality? Experimental tests of recognition, similarity-attraction, and consistency-attraction. J. Exp. Psychol. Appl..

[19] Robert LP (2020). A review of personality in human–robot interactions. Found. Trends Inf. Syst..

[20] Mairesse, F. & Walker, M. Personage: Personality generation for dialogue. In *Proceedings of the 45th Annual Meeting of the Association for Computational Linguistics*, 496–503 (2007).

[21] Holtgraves T (2011). Text messaging, personality, and the social context. J. Res. Pers..

[22] Yamada, K., Sasano, R. & Takeda, K. Incorporating textual information on user behavior for personality prediction. In *Proceedings of the 57th Annual Meeting of the Association for Computational Linguistics: Student Research Workshop*, 177–182 (2019).

[23] Pennebaker JW, King LA (1999). Linguistic styles: Language use as an individual difference. J. Pers. Soc. Psychol..

[24] Argamon, S., Dhawle, S., Koppel, M. & Pennebaker, J. W. Lexical predictors of personality type. In *Proceedings of the 2005 Joint Annual Meeting of the Interface and the Classification Society of North America*, 1–16 (2005).

[25] Mairesse F, Walker MA, Mehl MR, Moore RK (2007). Using linguistic cues for the automatic recognition of personality in conversation and text. J. Artif. Intell. Res..

[26] Yu, J. & Markov, K. Deep learning based personality recognition from facebook status updates. In *Proceedings of 2017 International Conference on Awareness Science and Technology*, 383–387 (IEEE, 2017).

[27] Tandera T, Suhartono D, Wongso R, Prasetio YL (2017). Personality prediction system from facebook users. Proced. Comput. Sci..

[28] Gjurković, M., Karan, M., Vukojević, I., Bošnjak, M. & Šnajder, J. PANDORA talks: Personality and demographics on Reddit. arXiv:2004.04460 (arXiv preprint) (2020).

[29] Khan AS (2020). Personality classification from online text using machine learning approach. Int. J. Adv. Comput. Sci. Appl..

[30] Gjurković, M. & Šnajder, J. Reddit: A gold mine for personality prediction. In *Proceedings of the Second Workshop on Computational Modeling of People’s Opinions, Personality, and Emotions in Social Media*, 87–97 (2018).

[31] Liu, Y. *et al.* RoBERTa: A robustly optimized BERT pretraining approach. arXiv:1907.11692 (arXiv preprint) (2019).

[32] Jiang, H., Zhang, X. & Choi, J. D. Automatic text-based personality recognition on monologues and multiparty dialogues using attentive networks and contextual embeddings (student abstract). In *Proceedings of the 34th AAAI Conference on Artificial Intelligence*, 13821–13822 (2020).

[33] Poria S, Majumder N, Mihalcea R, Hovy E (2019). Emotion recognition in conversation: Research challenges, datasets, and recent advances. IEEE Access.

[34] Hayati, S. A., Kang, D., Zhu, Q., Shi, W. & Yu, Z. Inspired: Toward sociable recommendation dialog systems. arXiv:2009.14306 (arXiv preprint) (2020).

[35] Fossati A, Borroni S, Marchione D, Maffei C (2011). The Big Five Inventory (BFI). Eur. J. Psychol. Assess..

[36] Radisavljević D, Rzepka R, Araki K (2023). Personality types and traits-Examining and leveraging the relationship between different personality models for mutual prediction. Appl. Sci..

[37] Furnham A (1996). The Big Five versus the Big Four: The relationship between the Myers-Briggs Type Indicator (MBTI) and NEO-PI five factor model of personality. Personal. Individ. Differ..

[38] Fang, Q. *et al.* On text-based personality computing: Challenges and future directions. arXiv:2212.06711 (arXiv preprint) (2022).

[39] De Raad B (2000). The Big Five Personality Factors: The Psycholexical Approach to Personality.

[40] Phan LV, Rauthmann JF (2021). Personality computing: New frontiers in personality assessment. Soc. Pers. Psychol. Compass.

[41] Zhang Z, Takanobu R, Zhu Q, Huang M, Zhu X (2020). Recent advances and challenges in task-oriented dialog systems. Sci. China Technol. Sci..

[42] Ni J, Young T, Pandelea V, Xue F, Cambria E (2022). Recent advances in deep learning based dialogue systems: A systematic survey. Artif. Intell. Rev..

[43] Pang Y (2019). A Pipeline-Based Task-Oriented Dialogue System on DSTC2 Dataset.

[44] Bordes, A., Boureau, Y.-L. & Weston, J. Learning End-to-End goal-oriented dialog. arXiv:1605.07683 (arXiv preprint) (2016).

[45] Wallace RS (2009). The anatomy of A.L.I.C.E. Parsing the Turing Test.

[46] Chen H, Liu X, Yin D, Tang J (2017). A survey on dialogue systems: Recent advances and new frontiers. ACM SIGKDD Explor. Newsl..

[47] Keh, S. S. & Cheng, I. Myers–Briggs personality classification and personality-specific language generation using pre-trained language models. arXiv:1907.06333 (arXiv preprint) (2019).

[48] Brodersen, K. H., Ong, C. S., Stephan, K. E. & Buhmann, J. M. The balanced accuracy and its posterior distribution. In *Proceedings of the 20th International Conference on Pattern Recognition*, 3121–3124 (2010).

[49] Amazon Mechanical Turk API reference. https://docs.aws.amazon.com/AWSMechTurk/latest/AWSMturkAPI/ApiReference_QualificationRequirementDataStructureArticle.html.

[50] Eric, M. *et al.* MultiWOZ 2.1: Multi-domain dialogue state corrections and state tracking baselines. arXiv:1907.01669 (arXiv preprint) (2019).

[51] Zhu, Q. *et al.* ConvLab-2: An open-source toolkit for building, evaluating, and diagnosing dialogue systems. In *Proceedings of the 58th Annual Meeting of the Association for Computational Linguistics*, 142–149 (2020).

[52] Hosseini-Asl, E., McCann, B., Wu, C.-S., Yavuz, S. & Socher, R. A simple language model for task-oriented dialogue. arXiv:2005.00796 (arXiv preprint) (2020).

[53] Louvan, S. & Magnini, B. Recent neural methods on slot filling and intent classification for task-oriented dialogue systems: A survey. arXiv:2011.00564 (arXiv preprint) (2020).

[54] Siddique A, Jamour F, Hristidis V (2021). Linguistically-enriched and context-awarezero-shot slot filling. Proc. Web Conf..

[55] Shuster, K., Smith, E. M., Ju, D. & Weston, J. Multi-modal open-domain dialogue. arXiv:2010.01082 (arXiv preprint) (2020).

[56] Miller, A. H. *et al.* ParlAI: A dialog research software platform. arXiv:1705.06476 (arXiv preprint) (2017).

[57] Zhang, S. *et al.* Personalizing dialogue agents: I have a dog, do you have pets too? In *Proceedings of the 56th Annual Meeting of the Association for Computational Linguistics*, 2204–2213 (2018).

[58] Singh, A. & Jain, G. Sentiment analysis of news headlines using simple transformers. In *Proceedings of the 2021 Asian Conference on Innovation in Technology*, 1–6 (2021).

[59] Kerz, E., Qiao, Y., Zanwar, S. & Wiechmann, D. SPADE: A Big Five-mturk dataset of argumentative speech enriched with socio-demographics for personality detection. In *Proceedings of the Thirteenth Language Resources and Evaluation Conference*, 6405–6419 (2022).

[60] He, P., Liu, X., Gao, J. & Chen, W. DeBERTa: Decoding-enhanced BERT with disentangled attention. arXiv:2006.03654 (arXiv preprint) (2020).

[61] Kanakaraddi, S. G. & Nandyal, S. S. Survey on parts of speech tagger techniques. In *2018 International Conference on Current Trends towards Converging Technologies (ICCTCT)*, 1–6 (IEEE, 2018).

[62] Boyd RL, Ashokkumar A, Seraj S, Pennebaker JW (2022). The Development and Psychometric Properties of LIWC-22.

[63] Eric, M. & Manning, C. D. Key-value retrieval networks for task-oriented dialogue. arXiv:1705.05414 (arXiv preprint) (2017).

[64] Hudeček, V. & Dušek, O. Are LLMs all you need for task-oriented dialogue?. arXiv:2304.06556 (arXiv preprint) (2023).

